# Upper Gastrointestinal Bleeding Due to Ruptured Gastric Varices: A Diagnostic Challenge

**DOI:** 10.7759/cureus.102939

**Published:** 2026-02-04

**Authors:** Luís Neves da Silva, Inês M Araújo, Rui Ribeiro, Sofia Teixeira, Francisco Mendes, Luis Flores, Joana Pereira

**Affiliations:** 1 Internal Medicine, Hospital de Braga, Braga, PRT; 2 Internal Medicine, Hospital Universitário de São João, Porto, PRT; 3 Gastroenterology, Hospital Universitário de São João, Porto, PRT

**Keywords:** acute upper gastrointestinal bleeding, chemotherapy-related toxicity, gastric varices, non cirrhotic portal hypertension, porto-sinusoidal vascular disorder

## Abstract

Gastric variceal (GV) bleeding is a life-threatening complication of portal hypertension, most commonly associated with cirrhosis. However, non-cirrhotic portal hypertension (NCPH) is an important and under-recognized cause, with portosinusoidal vascular disorder (PSVD) being a key entity, particularly in patients exposed to chemotherapy.

A 39-year-old man with a history of childhood neuroblastoma treated with dacarbazine, cyclophosphamide, doxorubicin, and vincristine presented with hematemesis and syncope. On admission, he was hypotensive and tachycardic. Laboratory tests revealed anemia (hemoglobin 6.9 g/dL), preserved liver function, and normal coagulation. Endoscopy showed a fundal GV with a “white nipple” sign. He received prompt vasoactive therapy, endoscopic cyanoacrylate injection, blood transfusion, and prophylaxis with ceftriaxone. Despite initial stabilization, bleeding recurred after terlipressin discontinuation, requiring a new endoscopic intervention. Common causes of cirrhosis were excluded. Imaging showed no parenchymal or vascular abnormalities. Elastography revealed normal liver stiffness (5.3 kPa) and elevated splenic stiffness (33.8 kPa). Hepatic angiography determined a portosystemic gradient of 15 mmHg. Liver biopsy demonstrated vascular changes consistent with PSVD. Extensive workup excluded other etiologies, leading to a final diagnosis of NCPH due to PSVD likely secondary to prior chemotherapy. The patient was started on a non-selective beta-blocker, with no further bleeding during follow-up.

This case highlights the diagnostic and therapeutic challenges of GV bleeding in NCPH and underscores the importance of a systematic and multidisciplinary workup to identify PSVD. A detailed history, including prior chemotherapy exposure, is crucial for appropriate management.

## Introduction

Gastric variceal (GV) bleeding is a rare but severe manifestation of portal hypertension (PH) and is associated with higher morbidity and mortality (30%) than esophageal varices [1,2]. GV develops due to increased portal pressure, most commonly due to cirrhosis. However, non-cirrhotic portal hypertension (NCPH) represents an important and under-recognized subset of patients [3].

NCPH encompasses a heterogeneous group of disorders with prehepatic, intrahepatic (non-cirrhotic), or posthepatic origins, unlike cirrhosis, where architectural distortion drives PH. NCPH is often linked to endothelial dysfunction, vascular remodeling, or idiopathic changes within the portal venous system. The term portosinusoidal vascular disorder (PSVD) has been introduced to unify conditions characterized by histological lesions in the sinusoids and small portal veins, in the absence of cirrhosis [4,5]. Liver biopsy is essential for diagnosis, revealing characteristic features with or without clinical PH. The increasing use of immunosuppressive and cytotoxic therapies for autoimmune and hematologic conditions has brought renewed attention to PSVD. Chemotherapy-induced vascular injury is a recognized but often delayed cause of PSVD, with latency periods that can span several years [6]. Other causes that have been associated with PSVD include myeloproliferative disorder, Hodgkin’s lymphoma, multiple myeloma, protein C or S deficiency, factor II or V gene mutation, antiphospholipid syndrome, common variable immunodeficiency, autoimmune hepatitis, systemic lupus erythematosus, scleroderma, rheumatoid arthritis, HIV infection, celiac disease, repeated gastrointestinal infections, Turner's syndrome, exposure to didanosine, azathioprine and tioguanine [7].

We herein report a case of upper gastrointestinal bleeding (UGIB) due to GV in a patient with remote childhood chemotherapy exposure, ultimately diagnosed with PSVD.

## Case presentation

A 39-year-old male presented to the emergency department with hematemesis and syncope. His medical history was significant for neuroblastoma diagnosed at the age of two, involving the adrenal gland, retroocular region, and sphenoid sinus. He was treated with dacarbazine, cyclophosphamide, doxorubicin, and vincristine, followed by left adrenalectomy. He remained under surveillance without recurrence.

**Figure 1:**
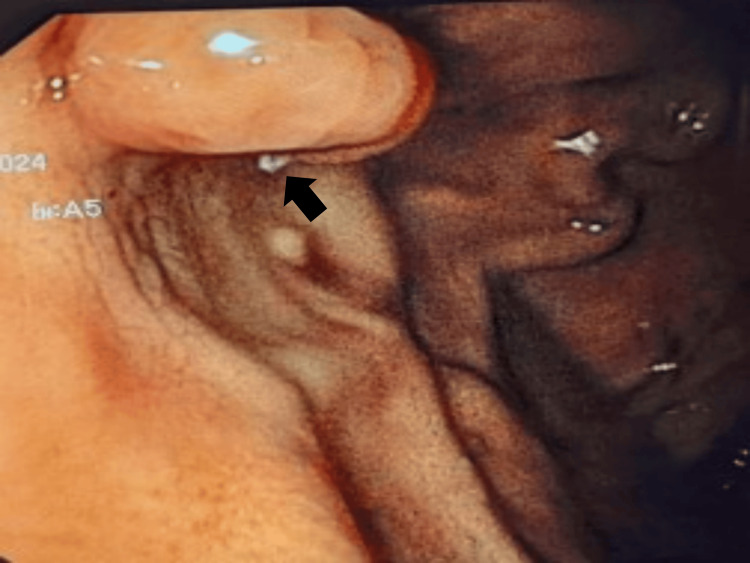
First endoscopic findings showing a gastric varix with a “white nipple” sign (arrow), indicating recent bleeding.

On admission, the patient was hypotensive and tachycardic. Laboratory tests revealed a hemoglobin of 6.9 g/dL and a platelet count of 236,000/uL. Liver enzymes and coagulation parameters were within normal limits. Upper gastrointestinal endoscopy identified a fundal GV with a “white nipple” sign, indicating recent bleeding (Figure 1).

Endoscopic therapy with cyanoacrylate injection, blood transfusions, terlipressin, and prophylactic ceftriaxone was performed. He was admitted to an intermediate care unit.

**Figure 2:**
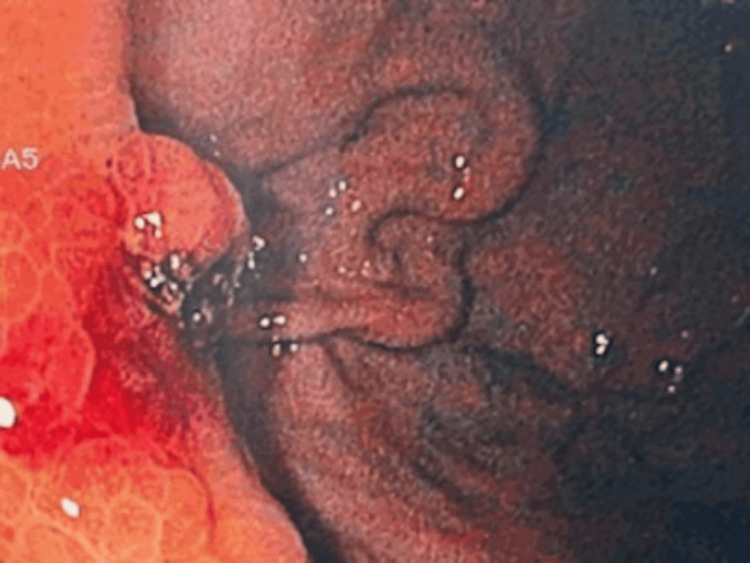
Second endoscopy findings. Endoscopic findings following the first recurrence reveal a gastric varix with hematic erosion, suggestive of recent bleeding, along with a scar consistent with a previous cyanoacrylate injection.

Despite initial stabilization, the patient experienced recurrent UGIB after discontinuation of terlipressin. Endoscopic therapy with cyanoacrylate was repeated (Figure 2), and Doppler endoscopic ultrasound confirmed the presence of GV. Vasoactive therapy with octreotide was subsequently restarted.

A comprehensive workup was done to determine the etiology of PH. Imaging studies, including contrast-enhanced computed tomography (CT) and magnetic resonance imaging (MRI), did not show parenchymal or vascular abnormalities. Hepatic and splenic elastography revealed a stiffness of 5.3 kPa and 33.8 kPa, respectively. Hepatic angiography showed no lesions and a portosystemic gradient of 15 mmHg. Liver biopsy showed preserved trabecular architecture of the liver with vascular alterations, namely marked dilation of venous-type vessels within the portal tracts, with focal herniation of these vessels into the adjacent parenchyma, as well as dilation of centrilobular veins and adjacent sinusoids. Serologies for the hepatitis C virus and HIV were negative, and hepatitis B virus immunity was confirmed. Autoimmune hepatitis markers were negative. Immunoglobulins A, G, and M, alpha-1 antitrypsin, and ceruloplasmin levels were normal. Antiphospholipid antibodies were negative, and protein C and S levels were normal. JAK2 V617F, CALR, and MPL mutations were negative. Celiac disease markers were also negative. Echocardiography was unrevealing. A diagnosis of NCPH due to PSVD was established, likely secondary to prior chemotherapy.

The patient was started on carvedilol, titrated up to 6.25 mg twice daily, and remained free of UGIB during follow-up.

## Discussion

This case illustrates the diagnostic and therapeutic challenges of GV bleeding in the context of NCPH, specifically due to PSVD secondary to remote chemotherapy exposure.

Diagnostic complexity in NCPH and PSVD

The patient presented with overt UGIB due to fundal GV, in the absence of advanced liver disease, biochemical liver dysfunction, or major vascular abnormalities. Imaging studies, including contrast-enhanced CT and MRI, revealed no parenchymal or vascular abnormalities. Hepatic elastography demonstrated normal stiffness (5.3 kPa), and laboratory tests did not show any liver dysfunction or damage. These findings, along with recurrent bleeding, elevated splenic stiffness (33.8 kPa), and a portosystemic gradient of 15 mmHg, supported NCPH as the cause for its clinical picture [8].

Histological evaluation was crucial for diagnosis. Liver biopsy revealed preserved lobular architecture with marked dilation of venous-type vessels within portal tracts, focal herniation into adjacent parenchyma, and centrilobular sinusoidal dilation - findings that are suggestive, though not specific, of PSVD [9].

Chemotherapy-induced vascular injury: a latent etiology

The patient’s history of neuroblastoma treated in early childhood with dacarbazine, cyclophosphamide, doxorubicin, and vincristine is notable. Although platinum-based agents have been the most implicated in PSVD, alkylating agents like cyclophosphamide have been implicated in endothelial injury and sinusoidal obstruction syndrome (SOS). While SOS typically presents acutely, the damage provoked by these chemotherapeutic agents can be subclinical, and delayed vascular remodeling may manifest years later as PSVD [6].

The latency of nearly four decades between chemotherapy and presentation is very long, but alongside the extensive negative study, it makes the chemotherapeutic exposure the most plausible etiology. It also reinforces the known insidious nature of chemotherapy-induced vascular injury and the importance of long-term surveillance in these patients.

Management considerations in GV bleeding with NCPH

The management of GV bleeding in NCPH follows principles similar to those in cirrhotic PH [10]. Acute management included cyanoacrylate injection, vasoactive therapy, and antibiotic prophylaxis. However, the early recurrence of bleeding upon cessation of terlipressin highlights the complexity in the management of GV and the need for sustained portal pressure control. Long-term therapy with carvedilol, a non-selective beta-blocker (NSBB), was initiated, reflecting actual recommendations. The patient’s favorable outcome, with no further UGIB during follow-up, supports the utility of NSBB in this setting. Transjugular intrahepatic portosystemic shunt is a therapeutic option for patients with refractory variceal bleeding, which was deferred in this case due to an effective response to medical and endoscopical therapy.

## Conclusions

In conclusion, GV bleeding in the context of NCPH and PSVD is a rare but serious condition. Clinicians should maintain a high index of suspicion for PSVD in patients with PH and no evidence of cirrhosis. A detailed history, including prior chemotherapy exposure, together with a systematic etiological workup to exclude common causes, is crucial. Early recognition and appropriate management can significantly improve patients' outcomes. A multidisciplinary approach involving internal medicine doctors, gastroenterologists, radiologists, and pathologists is essential.
